# Corrected data re-harvested: curating literature in the era of networked biodiversity informatics

**DOI:** 10.3897/BDJ.3.e4552

**Published:** 2015-01-21

**Authors:** Jeremy A. Miller, Teodor Georgiev, Pavel Stoev, Guido Sautter, Lyubomir Penev

**Affiliations:** † Naturalis Biodiversity Center, Leiden, Netherlands; ‡ www.Plazi.org, Bern, Switzerland; § Pensoft Publishers, Sofia, Bulgaria; | National Museum of Natural History and Pensoft Publishers, Sofia, Bulgaria; ¶ Institute of Biodiversity & Ecosystem Research, Bulgarian Academy of Sciences and Pensoft Publishers, Sofia, Bulgaria

## Main Text

Science makes progress through a constant process of re-evaluation. Revision and error correction are inevitable and generally healthy for the advancement of science. In biodiversity literature, re-evaluation of earlier work can lead to new conclusions, such as a revised taxonomic determination. When significant errors are discovered, conscientious authors may correct the record by publishing an erratum or corrigendum.

Aggregated global biodiversity data is an increasingly powerful resource supporting research, conservation, policy, and public bioliteracy (Hardisty et al. 2013, Arzberger et al. 2004). Along with databases devoted to specimen collections and observation records, literature is an integral part of the biodiversity informatics ecosystem (Miller et al. 2012, Penev et al. 2012, Penev et al. 2011a, Penev et al. 2011b). Pensoft journals pioneered the routine distribution of primary specimen data from publications to a collection of online resources, including the Global Biodiversity Information Facility (GBIF) and the Encyclopedia of Life (EOL) (Penev et al. 2009, Penev et al. 2008, Penev et al. 2010, Smith et al. 2013, Chavan and Penev 2011, Penev et al. 2012, Faulwetter et al. 2014). In the era of digital biodiversity informatics, maintaining data quality presents new challenges. In the realm of corrected taxonomic literature, we argue the objective should be to amend the structured digital record so that the correct information appears on resources like GBIF and the disavowed data are expunged. At the same time, good publishing practice requires that the original document and associated data remain part of the permanent scientific record.

A recent paper on central European spiders included a number of taxonomic errors (Čandek et al. 2013). In a corrigendum published in this issue (Čandek et al. 2015), the authors duly correct the record. Data from the original publication have already been harvested by online resources including GBIF. To guarantee that the data is corrected not only in the scientific literature but also in GBIF, the Darwin Core Archive (DwC-A) file (which is the vehicle for distributing content to a collection of online resources; GBIF 2010, Wieczorek et al. 2012) has been updated and submitted to GBIF. The supplier (Pensoft) needs to trigger a re-indexing through the API (Application Programming Interface, a set of protocols that, in this context, is used to share data between software applications) and the content will be added to the indexing queue. Normally it takes few hours to be indexed (Markus Döring, GBIF senior software developer, pers. comm.). However, the original DwC-A file remains available for users to download from the journal web site. The original and corrected data files are clearly labeled as such and visible alongside the original publication. A link landing at the corrigendum will be added to the original publication metadata to facilitate its discoverability. In addition, the XML data file from the original article has been retained on the servers of Plazi, but the XML tags have been amended to render them no longer exposed for harvest. A modified XML document combining the original data with all corrections specified in the corrigendum (i.e., a single corrected document) has been made available as a supplementary document linked to the corrigendum, and will be uploaded to Plazi upon publication of the corrigendum. This will present the corrected data in XML form, permiting the export of treatments and data to various aggregators (Penev et al. 2012).

This demonstrates a small but important step toward insuring high data quality in the era of growing online networks of biodiversity data. The power of structured biodiversity data aggregated from many sources and freely available online is becoming increasingly valuable to a range of traditional and nontraditional data consumers (Moritz et al. 2011, Arzberger et al. 2004). It is in the interest of the general community and publishers in particular to insure that data are of the highest possible standard.

As large aggregations of data become increasingly important in myriad scientific disciplines, warnings are being sounded that the Achilles' heel of these otherwise promising enterprises is data quality. Big data need robust curatorial mechanisms to assure accuracy and reliability so that the promise of these great collaborative efforts is not squandered (Leonelli 2014, Mesibov 2013, Thessen and Patterson 2011, Hjarding et al. 2014, Belbin et al. 2013). An emerging solution is aimed at collections data from natural history research institutions, a major class of data suppliers to GBIF (Berendsohn et al. 2010, Robertson et al. 2014). The idea is to provide a mechanism for users to flag suspicious records and make possible errors known to data providers (who have the power to check and correct errors) and the broader user community (Wang et al. 2009, Tschöpe et al. 2013, Morris et al. 2013). Wide online access to primary biodiversity data through aggregating databases like GBIF facilitate unprecedented power for data comparison and scrutiny, well beyond what is possible with unnetworked collections databases and literature published on paper without structured digital data. Errors are inevitable in any field, but science is a self-correcting process. The path forward toward well-curated, accessible, aggregated biodiversity data can be accomplished with the participation of the whole community, including publishers, authors, institutional collections personnel, and end users.
